# Clinical significance of polyunsaturated fatty acids in the prevention of cardiovascular diseases

**DOI:** 10.3389/fnut.2022.998291

**Published:** 2022-10-06

**Authors:** Stanislav Kotlyarov, Anna Kotlyarova

**Affiliations:** ^1^Department of Nursing, Ryazan State Medical University, Ryazan, Russia; ^2^Department of Pharmacy Management and Economics, Ryazan State Medical University, Ryazan, Russia

**Keywords:** atherosclerosis, cardiovascular disease, polyunsaturated fatty acids, inflammation, lipid metabolism

## Abstract

Cardiovascular diseases are one of the most important problems of modern medicine. They are associated with a large number of health care visits, hospitalizations and mortality. Prevention of atherosclerosis is one of the most effective strategies and should start as early as possible. Correction of lipid metabolism disorders is associated with definite clinical successes, both in primary prevention and in the prevention of complications of many cardiovascular diseases. A growing body of evidence suggests a multifaceted role for polyunsaturated fatty acids. They demonstrate a variety of functions in inflammation, both participating directly in a number of cellular processes and acting as a precursor for subsequent biosynthesis of lipid mediators. Extensive clinical data also support the importance of polyunsaturated fatty acids, but all questions have not been answered to date, indicating the need for further research.

## Introduction

Cardiovascular diseases are one of the most important problems of modern medicine (1). Coronary heart disease, ischemic stroke, and peripheral arterial diseases are clinical manifestations of atherosclerosis of different vascular locations. The increasing prevalence, medical, social and economic consequences of these diseases require new approaches aimed at improving the efficiency of early diagnosis and treatment (2–4). Indeed, atherosclerosis develops over many years, but patients may stay out of the doctor’s sight all this time, as a result, they often first seek medical care only at clinically advanced stages. Atherosclerosis is considered to be a disease whose development is strongly associated with risk factors, such as eating disorders, overweight and obesity, physical inactivity, and smoking (5–7). These modifiable risk factors are an important therapeutic target, which should be treated as early as possible (8). Lipid metabolism disorders play a key role in atherogenesis, and their correction is the basis of drug and non-drug therapy for these patients (9, 10).

A growing body of evidence has made it possible to determine the leading role of impaired lipid metabolism in many processes occurring in the vascular wall in atherosclerosis (11). Fatty acids are essential molecules that exhibit a variety of functions. Because of their diversity, they are involved in a variety of processes.

Depending on the length of the carbon chains, fatty acids are commonly classified as short-chain (have less than 6 carbon atoms), medium-chain (6–12 carbon atoms) and long-chain (more than 12 carbon atoms). In addition, fatty acids are divided by the degree of saturation of the carbon chain with hydrogen atoms. This classification distinguishes saturated fatty acids (SFAs), monounsaturated fatty acids (MUFAs) and polyunsaturated fatty acids (PUFAs). Of clinical importance is another classification that distinguishes ω-3 PUFAs with the end double bond at C3, counting from the methyl end of the hydrocarbon chain, and ω-6 PUFAs with the end double bond at C6.

Clinical interest in PUFAs increased in the 1970s after a series of epidemiological studies were published showing that Greenland Eskimos had a lower risk of coronary heart disease and diabetes compared to the Danish population, which was linked to a high consumption of ω-3 PUFAs derived from marine fish, seals and whales (12, 13). It was shown that the fatty acid structure consumed by the Greenland Eskimos hunters was characterized by a higher content of long-chain polyunsaturated fatty acids (especially eicosapentaenoic acid and docosahexaenoic acid) and a lower content of linoleic and linolenic acids (14). This corresponded to lower plasma concentrations of triglycerides and very low-density lipoproteins in Eskimos than in Western populations (14). Although total polyunsaturated fatty acid concentrations were lower in Greenland Eskimos than in other groups, a higher proportion of palmitic, palmitoleic and eicosapentaenoic acids and a lower concentration of linoleic acid were found in Greenland Eskimos plasma (15).

Japanese people living on Kohama Island in Okinawa have a very low incidence of cardiovascular disease, which is thought to be due to a high intake of eicosapentaenoic and docosahexaenoic acids from fresh fish. In a study of serum from elderly people from this population, higher levels of total eicosapolyenoic acids were found than in mainland people. In addition, a positive correlation was found between serum eicosapentaenoic acid concentrations and high-density lipoprotein (HDL) concentrations (16). Consumption of seafood, a source of eicosapentaenoic and docosahexaenoic acids, was associated with low rates of coronary heart disease mortality in the Inuit population of Nunavik (17).

**FIGURE 1:**
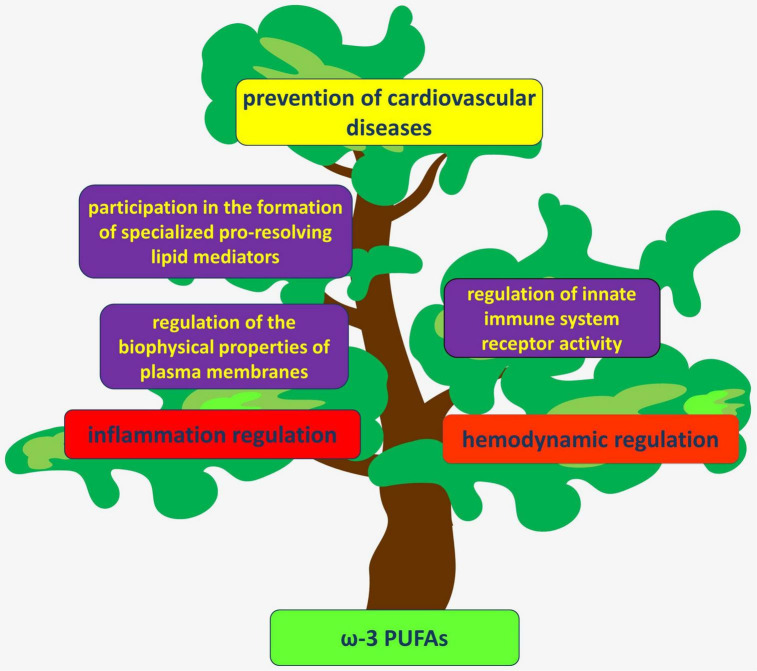
PUFAs act through several mechanisms to regulate hemodynamics and inflammation in the vascular wall. Their anti-inflammatory and atheroprotective effects are considered beneficial for the prevention of various cardiovascular diseases.

These and other findings have increased attention to the possible therapeutic value of PUFAs for cardiovascular disease (Figure 1).

Marine fish is a valuable source of polyunsaturated fatty acids. The CALIPSO study showed that eel, salmon, swordfish, and halibut are high in ω-3 PUFAs such as eicosapentaenoic, docosahexaenoic and docosapentaenoic fatty acids (18). In addition, antarctic krill oil was also shown to contain eicosapentaenoic acid and docosahexaenoic acid, which account for over 27% of total fatty acids (19).

### Molecular mechanisms in which polyunsaturated fatty acids are involved

**FIGURE 2:**
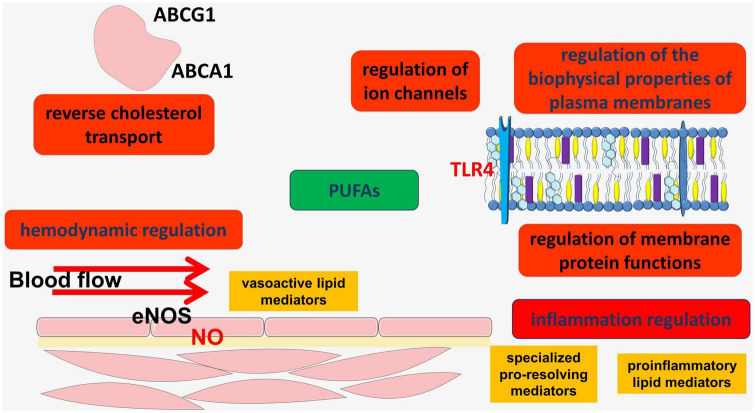
Participation of PUFAs in the regulation of some processes in the vascular wall.

Polyunsaturated fatty acids (PUFAs) are important participants in many physiological processes and are associated with the regulation of inflammation, antioxidant protection, the regulation of vascular hemodynamics and other important biological functions (Figure 2).

### Participation of polyunsaturated fatty acids in the regulation of inflammation

#### Participation of polyunsaturated fatty acids in formation of lipid mediators

**FIGURE 3:**
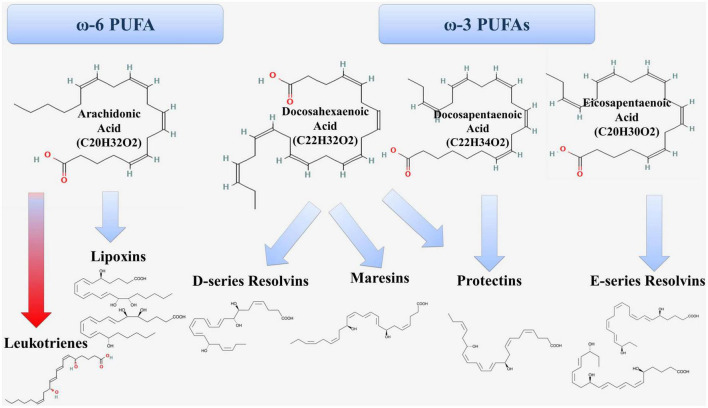
Formation of proinflammatory (red arrow) and anti-inflammatory (blue arrow) lipid mediators from PUFAs.

According to modern concepts, atherosclerosis is an inflammatory disease of arterial intima, in which the balance between the mechanisms of activation and resolution of inflammation is disturbed. PUFAs are substrates for the synthesis of both proinflammatory and specialized pro-resolving lipid mediators (Figure 3) (20).

Arachidonic acid is ω-6 PUFA and is metabolized through the lipoxygenase (LOX) pathway, the cyclooxygenase (COX) pathway and the cytochrome P450 pathway (20). In the lipoxygenase pathway, arachidonic acid is a substrate for the formation of proinflammatory leukotrienes and pro-resolving lipoxins (Figure 1). The 5-LOX enzyme is at the crossroads of the proinflammatory and specialized pro-resolving lipid mediators synthesis pathways (21).

Leukotrienes play an important role in inflammation in atherosclerosis (22, 23). They are mainly produced by macrophages that infiltrate atherosclerotic lesions. Leukotriene (LT) B4 is a potent chemoattractant for monocytes, neutrophils, and lymphocytes. It participates in atherogenesis by promoting leukocyte adhesion to the endothelium, increasing vascular permeability, and promoting proliferation and migration of vascular smooth muscle cells (VSMCs) (24, 25).

Lipoxins belong to the group of specialized pro-resolving lipid mediators and play an important role in atheroprotection. Lipoxin A4 (LXA4) and lipoxin B4 (LXB4) as well as their epimers such as 15-epi-LXA4 and 15-epi-LXB4 are currently known. The formation of lipoxin epimers is related to the action of aspirin on COX-2, leading to the formation of 15R-hydroxyecosatetraenoic acid (15R-HETE), which is then metabolized by 5-LOX to form epi-lipoxins (26). This pathway involves intercellular interactions between leukocytes and endothelial cells (27).

LXA4 and LXB4 show multiple anti-inflammatory effects. They inhibit leukotriene B4-stimulated transendothelial migration of neutrophils and promote the uptake of apoptotic neutrophils (28) by macrophages (29–31). LXA4 has an inhibitory effect on NF-κB, on interleukin (IL) -1β, IL-6, tumor necrosis factor-α (TNF-α) expression, while enhancing IL-10 expression and improving hemodynamic indices, myocardial structure and function (32). 15-epi-lipoxin A4, acting through endothelial nitric oxide synthase (eNOS) and inducible nitric oxide synthase (iNOS) promotes NO production, negatively regulating leukocyte-endothelial interaction (33).

The cyclooxygenase pathway of arachidonic acid metabolism promotes the formation of prostaglandins (PGE2, PGF2, PGD2), thromboxane (TxA2) and prostacyclin (PGI2). Prostaglandins may be involved in inflammation in the vascular wall (34). PGE2 acts through its receptors and induces expression of matrix metalloproteinase, which may contribute to the instability of atherosclerotic plaques (35). PGE2 also demonstrates hemodynamic actions, which are mediated by complex interactions of several receptor subtypes (36, 37).

Thus, arachidonic acid is a precursor for the formation of lipid mediators involved in both the maintenance and resolution of inflammation.

Eicosopentaenoic acid (EPA, 20:5 ω-3) is a ω-3 PUFA and is the precursor for the formation of E-series resolvins (Figure 3). RvE1 has anti-inflammatory and pro-resolving effects through several mechanisms, including inhibition of the release of inflammatory mediators (38, 39) and activation of the process of efferocytosis of apoptotic neutrophils by macrophages (40, 41). In experimental animal models, RvE1 administration suppressed atherogenesis (42). RvE1 had a local anti-inflammatory effect in the aorta of mice and reduced the area and severity of atherosclerotic lesions without affecting plasma lipids (43).

Docosahexaenoic acid (DHA, 22: 6 ω-3) is also ω-3 PUFA and serves as a precursor for the formation of D-series resolvins, protectins, and maresins (Figure 3). RvD1 inhibits the release of proinflammatory cytokines (IL-6 and TNF-α) (44–46) and enhances the production of anti-inflammatory cytokines in macrophages. In addition, RvD1 affects the stability of the atherosclerotic plaque, promotes the reduction of necroptotic cells, activates the PI3K/Akt pathway, which reduces the negative effects of ischemia and reduces the infarct size (47). Another member of this series of resolvins, RvD2 is involved in the regulation of nitric oxide production as well as modulation of leukocyte adhesion receptor expression, which reduces leukocyte-endothelial interaction (48). In turn, protectin PD1 has antiapoptotic and anti-inflammatory activity. It was shown that immediately after the onset of myocardial infarction the levels of protectins increased, which is part of the early anti-inflammatory response after ST-elevation myocardial infarction (49).

Maresins are formed in macrophages from docosahexaenoic acid and have anti-inflammatory and reparative effects (50). The mechanism of anti-inflammatory action of maresins is to decrease the levels of inflammatory mediators such as IL-6, IL-1β, TNF-α (51, 52) and to increase the production of anti-inflammatory mediators (IL-10) (53, 54). In addition, maresins contribute to the enhancement of phagocytosis and efferocytosis by macrophages and help to limit the infiltration of polymorphonuclear leukocytes (55–60).

Fatty acid esters of hydroxy fatty acids (FAHFAs) represent another class of endogenous lipids with various biological effects (61). The hydroxy fatty acid esters of docosahexaenoic acid (FAHFAs) have anti-inflammatory properties (62). 13-DHAHLA (13-docosahexaenoic acid hydroxy linoleic acid) exhibits anti-inflammatory and pro-resolving properties by reducing lipopolysaccharide-induced macrophage activation and enhancing phagocytosis of zymosan particles (62). At the same time, levels of 13-DHAHLA correlated with the patients’ serum omega-3 index (62). 12-EPAHSA (eicosapentaenoic acid esterified 12-hydroxy stearic acid) increased the expression of Nrf2-dependent antioxidant enzyme genes such as NQO1, GCLM, GCLC, SOD-1 and HO-1 (61).

Thus, PUFAs are an important source of both proinflammatory and anti-inflammatory mediators, the balance of which is important for atherogenesis.

#### Regulation of the innate immune system

The innate immune system plays an important role in atherogenesis. As part of an evolutionarily ancient system of identification and response to pathogen-associated molecular patterns (PAMP), the innate immune system relies on a large family of pattern recognition receptors (PRRs). These receptors, whose role is well known in atherogenesis, include Toll-like receptor 4 (TLR4), which detects the lipopolysaccharide of Gram-negative bacteria. The TLR4 signaling pathway is closely related to lipid rafts, where TLR4 is recruited upon activation. Fatty acids can influence these processes. It was shown that saturated lauric acid induced dimerization and recruitment of TLR4 to lipid rafts (63). In contrast to saturated fatty acids, unsaturated fatty acids did not activate TLR4 (64). In contrast, docosahexaenoic acid suppressed NF-κB activation and COX-2 expression induced by TLR agonist 2, 3, 4, 5, or 9 in a macrophage cell line (RAW264.7) (65). Thus, docosahexaenoic acid can inhibit TLR4 activation induced by saturated fatty acids. In addition, it was shown that by binding to MD2 (which is a TLR4 co-receptor), arachidonic acid can prevent its association with lipopolysaccharide (LPS), reducing LPS-induced inflammation (66).

It also seems important that while saturated fatty acids can promote NLRP3 inflammasome activation, PUFAs inhibit its activity (67). Dietary PUFAs reduced LPS-induced IL-1β secretion in mice *in vivo* (68). This is important given the data on the role of NLRP3-inflosome and IL-1β in the pathogenesis of atherosclerosis (69, 70).

### Other anti-inflammatory mechanisms

It has been shown that eicosapentaenoic acid can decrease iNOS activity (71). In addition, in mouse macrophages stimulated with interferon-γ plus LPS, docosahexaenoic acid inhibited iNOS mRNA accumulation (72). This seems interesting given the role of macrophage iNOS in the overproduction of NO, which in high concentrations can have cytotoxic effects.

### Participation of polyunsaturated fatty acids in hemodynamic regulation

One of the most important advances in vascular biology has been the understanding that endothelial cells can detect changes in blood flow and participate in its regulation through the production of vasoactive substances. One of the key molecules involved in vasodilation is nitric oxide. Nitric oxide exhibits several functions in the vascular wall and is produced by endothelial cells via eNOS. Deficiency in NO production is considered an important marker of endothelial dysfunction, which plays a key role in atherogenesis. eNOS localizes in caveolae, specific structural and functional lipid domains of the plasma membrane. Studies emphasize the important role of endothelial caveolae in the regulation of eNOS activation. In an experiment on rats, it has been shown that destruction of caveolae leads to a decrease in endothelial NO release (73).

It was found that fatty acids can affect the bioavailability of nitric oxide (74). Eicosapentaenoic acid-treated cells increase NO production while decreasing ONOO- release. Cell exposure to docosahexaenoic acid increased NO production but had no effect on ONOO- release. In turn, exposure to arachidonic acid had no effect on NO and ONOO- release (74).

Eicosapentaenoic acid and docosahexaenoic acid (6:1) were shown to induce phosphorylation of Src, Akt, and eNOS in Ser 1177 and induce endothelium-dependent NO-mediated relaxation in pig coronary artery in the experiment (75).

In addition to NO, lipid mediators derived from PUFAs play an active role in the regulation of vascular hemodynamics. The crosslinks between hemodynamics and lipid mediators are due to the fact that shear stress can activate cytosolic phospholipase A2, which promotes the release of arachidonic acid from phospholipids of the plasma membrane (76). It is also suggested that NO can enhance COX2 activity through S-nitrosylation, resulting in increased prostaglandin production (77). PGI2 is formed from arachidonic acid and promotes relaxation of VSMCs, resulting in a marked vasodilator effect.

In addition, arachidonic acid is metabolized by cytochrome P450s to hydroxyeicosatetraenoic acids (HETEs) and epoxyeicosatrienoic acids (EETs). 20-HETE is formed by ω-hydroxylation of arachidonic acid in VSMCs and is a potent vasoconstrictor (78, 79). 20-HETE acts on ion channels (79, 80). The G-protein receptor 75 (GPR75) has been identified as a target for 20-HETE and mediates its involvement in the development of hypertension (81, 82). 20-HETE has been shown to uncouple eNOS and reduce NO production and to stimulate NF-κB activation and inflammatory cytokine production in human endothelial cells (83, 84). In turn, 20-HETE formation in vascular smooth muscle is inhibited by nitric oxide (85–87). This is because NO can inhibit heme-containing proteins such as CYP (88). Elevated 20-HETE causes endothelial dysfunction and impairs coronary collateral growth in the metabolic syndrome (89). Urinary excretion of 20-HETE has also been shown to be associated with endothelial dysfunction (90).

Endothelial cells in the renal, cerebral, and coronary arteries produce EET from arachidonic acid (80, 91–95). They have vasodilators and are known as endothelium-derived hyperpolarizing factor (EDHF), which hyperpolarizes vascular smooth muscle cells (VSMCs) by activating large-conductance calcium-activated K + channels (79, 93, 96). Thus, ω-3 PUFAs enhance endothelium-dependent vasorelaxation through enhanced release of EDRF and vasodilatory prostaglandins (97). 20-HETE has been shown to inhibit and EETs to activate renal microvascular smooth muscle cell large-conductance calcium-activated K + channels (80).

Interestingly, an analysis of the prognostic impact of PUFA metabolites on clinical outcomes in coronary heart disease showed that the incidence of future myocardial infarction was more frequent in patients with higher baseline levels of 8- HETE, 9-HETE, 11-HETE, 12-HETE, 15-HETE, 19-HETE, 20-HETE, 5,6-EET, 8,9-EET, 11,12-EET and 14-15-EET (98).

In another study it was shown that prostanoids derived from EPA exerts an endothelium-independent vasorelaxant effect in the isolated aorta of Wistar Kyoto (WKY) rats by activating ATP-sensitive potassium channels (K_*ATP*_) (99). Docosahexaenoic acid can be metabolized to epoxidocosapentaenoic acids *via* CYP450, which can activate K_*ATP*_ at high concentrations of docosahexaenoic acid, whereas low concentrations act through Ca2 + -mediated modulation (100). EPA can also directly modulate intracellular Ca2 + signaling in VSMCs, which contributes to a vasodilatory effect (101).

Thus, ω-3 PUFAs exhibit endothelium-independent vasodilatory effects that are associated with the opening of large conductance calcium-activated potassium channels, K_*ATP*_ and members of the Kv7 family of voltage-gated potassium channels in VSMCs (102). The resulting effect is hyperpolarization and relaxation of VSMCs and, consequently, a vasodilatory effect (102).

### Role of polyunsaturated fatty acids in the biophysics of plasma membranes

Fatty acids that are part of plasma membrane lipids are involved in the regulation of membrane biophysical properties and physiological functions (103). Chain length and degree of unsaturation are important factors determining the role of ω-3 PUFAs in cell membranes. The lipid bilayer of the plasma membrane has a complex structural organization in which the leading role is assigned to the spatial orientation of cholesterol molecules as well as the fatty acid tails of phospholipids, whose unsaturation can significantly influence such biophysical properties as fluidity (104). At the same time, the biophysical properties of plasma membranes provide functions for many membrane proteins (105, 106). Lipid ordering is known to affect the possibility of necessary conformational changes in proteins to perform their functions. Fatty acids in membrane phospholipids are involved in maintaining the biophysical properties of plasma membranes, providing, on the one hand, optimal fluidity to allow proteins to perform the conformational changes required for their function and, on the other hand, providing sufficient viscosity, which is required for their membrane localization (107, 108). Phospholipid tails of saturated fatty acids maintain lipid organization, whereas phospholipids containing polyunsaturated fatty acids are significantly more disordered. ω-3 PUFAs are incorporated into phospholipids of lipid domains, displacing cholesterol and affecting the molecular order of lipid microdomains (109). This is due to the fact that PUFAs chains have many rapidly changing conformations that push away the rigid steroid moiety of cholesterol molecule, affecting the lipid ordering of the membrane (109, 110). Meanwhile, unordered PUFA-rich domains coexist with highly ordered lipid rafts enriched in sphingolipids and cholesterol (111). The incorporation of ω-3 PUFAs into phospholipids of plasma membranes has enormous potential to change the organization of their molecular architecture, to remodel lipid-protein interactions and the functions of membrane proteins (109).

It was shown that eicosapentaenoic acid changes the lipid composition in caveolae and induces eNOS translocation from caveolae to soluble fractions, which was accompanied by a stimulated ability to produce NO in cells (112). In turn, docosahexaenoic acid changes the microenvironment of caveolae not only by changing the lipid composition of the membrane but also by changing the distribution of basic structural proteins, such as caveolin-1, and also promotes eNOS displacement from caveolae (113). Moreover, treatment of cells with docosahexaenoic acid significantly increases eNOS activity compared to the control (113). In addition, docosahexaenoic acid was shown to enhance eNOS and Akt activity, increase HSP90 (heat shock protein 90) expression, and increase NO bioavailability in response to Akt-kinase activation (114).

Interestingly, PUFAs can have different effects on the lipid organization of membranes, depending on the chain length of the fatty acids and the degree of unsaturation. Eicosapentaenoic acid largely avoids interactions within lipid rafts and prefers a disordered non-raft region, whereas docosahexaenoic acid can be incorporated lipid rafts, modifying their biophysical organization (115). Eicosapentaenoic acid has been shown to decrease membrane fluidity, inhibit cholesterol domain formation, and normalize bilayer width in atherosclerotic-like model membranes (116). At the same time, docosahexaenoic acid had no effect on membrane width, but induced formation of cholesterol domains and increased fluidity of the model membrane (116). Thus, exposure to docosahexaenoic acid may increase the fluidity of the plasma membrane of endothelial cells to a greater extent than eicosapentaenoic acid (117). This effect may be related to the greater ability of docosahexaenoic acid to reduce membrane cholesterol content or the molar ratio of cholesterol to phospholipids (117). Thus, eicosapentaenoic acid and docosahexaenoic acid can mutually counter-regulate each other’s physiological effects in different tissues (115).

Docosahexaenoic acid has 22 carbon atoms and 6 double bonds, being the most unsaturated and one of the longest ω-3 PUFAs, thus having a significant effect on the biophysical properties of plasma membranes (110). It has been shown that docosahexaenoic acid molecules can spontaneously assemble into nanoclusters and localize in liquid-disordered membrane domains (118). Docosahexaenoic acid-rich membranes are fluid, have increased permeability, and are thin, due to the high disorder of the acyl chains and their reduced effective length (110). In addition, docosahexaenoic acid makes simulated bilayer phospholipids less sensitive to applied surface tension than saturated phospholipids, which, suggests a decrease in membrane elasticity, i.e., area elastic modulus, bending rigidity (119). Most tissues typically contain docosahexaenoic acid well below 5 mol% of the total acyl chains, but this amount increases with PUFA supplementation (110).

Numerous data indicate the effect of PUFAs on cardiac ion channels, which affects the occurrence of arrhythmias. Moreover, the effect of PUFAs on the biophysical properties of ion channels and cardiac electrophysiology may differ significantly during acute and long-term administration of PUFAs, when they are gradually incorporated into plasma membranes (120). It was found that acute administration of ω3-PUFAs reduces the cardiac sodium current (I_*Na*_), which corresponds to a reduction in excitability and a slowing of ventricular conduction, whereas membrane incorporated ω-3 PUFAs do not alter INa (121–127). The ability of docosahexaenoic acid, eicosapentaenoic acid and alpha-linolenic acid to block cardiac sodium currents was found to correlate with their ability to increase membrane fluidity of isolated adult rat ventricular myocytes (123).

In addition, acute administration of ω-3 PUFAs to ventricular myocytes reduced voltage-gated L-type calcium currents (I_*Ca,L*_), lowering the plateau of the action potential (128, 129). At the same time, ω-3 PUFAs incorporated into the sarcolemma also reduce I_*Ca,L*_ and shortened pig ventricular action potentials (126). Spontaneous calcium release from the sarcoplasmic reticulum, which underlies after-depolarization associated arrhythmias in heart failure, was also found to be reduced after acute administration of eicosapentaenoic acid to isolated ventricular myocytes in rabbits with heart failure (120, 130, 131). The inhibitory effect of ω-3 PUFAs on cardiac Ito (transient outward potassium current) and IK (delayed rectifier potassium current) was also shown to be less effective compared with their effect on cardiac Na and Ca currents (132).

These data indicate a significant effect of PUFAs on cell membrane biophysical properties and cardiac electrophysiology. However, acute and long-term administration to PUFAs differs. At the same time, PUFAs may have both pro- and antiarrhythmic effects (126).

Thus, ω-3 PUFAs, when incorporated into membrane phospholipids, may influence the biophysical properties of membranes and the function of membrane proteins, which, in addition to their role as lipid mediators, is a promising area for future research. The extensive data available to date demonstrate the multifaceted role of PUFAs in many physiological and pathological processes. As has been shown in many studies ω-3 PUFAs demonstrate predominantly atheroprotective effects, which formed the basis for evaluating their preventive and therapeutic efficacy. Dietary and circulating eicosapentaenoic acid and docosahexaenoic acid have been shown to be inversely associated with the incidence of cardiovascular disease. No significant associations with cardiovascular disease were observed for alpha-linolenic acid and ω-6 PUFAs (linoleic acid, arachidonic acid) (133).

### Participation of polyunsaturated fatty acids in other processes

Disorder of reverse cholesterol transport is considered to be an important part of the pathogenesis of atherosclerosis. A large number of data indicate a role of ABCA1 and ABCG1 in the regulation of cholesterol transport from macrophages and HDL formation. A decrease in the expression and functional activity of these transporters is associated with the formation of “foam cells” and the progression of atherosclerosis. Unsaturated fatty acids such as palmitoleic, oleic, and linoleic acids have been shown to reduce ABCA1 expression at transcriptional as well as posttranscriptional levels, reducing cholesterol efflux (134, 135). Eicosapentaenoic and oleic acids also reduced ABCA1 promoter activity in macrophages. Unsaturated fatty acids also suppressed ABCG1 gene expression through a mechanism that involves LXR/RXR binding to promoters (136). In addition, inclusion of eicosapentaenoic acid in the membrane impairs ABCA1-dependent cholesterol efflux through the protein kinase A signaling pathway in primary human macrophages (137). Inclusion of eicosapentaenoic acid in the membrane impairs cholesterol efflux from cholesterol-laden human macrophages also by reducing the mobilization of cholesterol ester from lipid droplets (138). On the other hand, incorporation of eicosapentaenoic acid into the membranes increased ABCA1 functionality in cholesterol normal human THP-1 macrophages, probably through a combination of different mechanisms (139). In addition, dietary fish consumption and ω-3 PUFAs were associated with levels of ABCA1 leukocyte DNA methylation (140).

Interestingly, the heart tissue of mice on a high-fat diet (HFD) showed an increase in arachidonic, linoleic, and docosahexaenoic acids and a decrease in eicosapentaenoic acid (141). The incorporation of PUFAs into the cell membrane can change the biophysical properties of the membrane and affect cardiac function (141).

Linoleic acid is a biochemical precursor of arachidonic acid, which is used to synthesize proinflammatory lipid mediators (142). Linoleic acid can be converted to arachidonic acid, and α-linolenic acid can be metabolized to eicosapentaenoic acid and docosahexaenoic acid (143, 144). In addition, linoleic acid is a substrate for the synthesis of bioactive oxidized metabolites such as 9- and 13-hydroxyoctadecadienoic acid (9- and 13-HODE), which may be involved in various processes related to atherogenesis (145–147). We found that the level of 9-HODE obtained from LDL of young patients with atherosclerosis was increased 20-fold compared with samples taken from healthy individuals (146). 9-HODE and 13-HODE stimulate the release of IL-1β from macrophages (148). In addition, linoleic acid exposure increases the transport of human LDL through cultured endothelial monolayers (149).

Atheroma samples were shown to contain a higher proportion of linoleic acid (150). Consumption of more linoleic acid increases its amount in complicated aortic plaques (151). On the other hand, 13-HODE was found to induce cholesterol efflux from macrophages through the PPAR-LXRa-ABCA1/SR-BI pathway (152).

Thus, PUFAs are involved in various physiological and pathological processes, whose complex relationships may be disturbed during atherogenesis.

## Clinical significance of polyunsaturated fatty acids

Accumulating evidence suggests a potential benefit of ω-3 PUFAs in the prevention of cardiovascular disease. Fish oil supplementation in healthy volunteers was associated with improved endothelial function and decreased resting heart rate (153). In addition, ω-3 PUFA supplementation significantly improved endothelial function and reduced inflammatory markers in the offspring of patients with type 2 diabetes (154). Dietary supplements containing marine ω-3 PUFAs improved endothelium-dependent dilatation of large arteries in patients with hypercholesterolemia but had no effect on endothelium-independent dilatation (155). Fish oil supplements increased the number of endothelial progenitor cells and decreased the number of endothelial microparticles, potentially contributing to endothelial integrity (156).

A randomized, placebo-controlled trial including 53 mildly hypertriacylglycerolemic subjects evaluated the potential benefit of daily ω-3 PUFA supplementation for improving cardiovascular health. After 10 weeks, subjects taking ω-3 PUFAs at 3 grams per day had significantly increased ω-3 PUFA levels in plasma and erythrocytes. In addition, the EPA-derived mediators PGE3, 12- HEPE, 15- HEPE, and 18-HEPE increased in plasma. This was consistent with improvements in cardiovascular risk factors such as HDL levels, triacylglycerides, arachidonic acid/eicosapentaenoic acid ratio, and ω-3 index (157).

Results from a recent pooled analysis of 4 international cohort trials including 191,558 individuals from 58 countries showed that a minimum fish consumption of 175 grams (approximately 2 servings) per week was associated with a lower risk of major cardiovascular disease and mortality among patients with prior cardiovascular disease, but not in the general population (158).

A comprehensive review of 34 meta-analyses of prospective cohort studies on the association of fish consumption and the risk of cardiovascular disease found that an increase in fish consumption of 100 grams per day was associated with a lower risk of all-cause mortality (summary relative risk (SRR): 0.92; 95% confidence interval (CI): 0.87, 0.97), cardiovascular mortality (SRR: 0.75; 95% CI: 0.65, 0.87), risk of coronary heart disease (SRR: 0.88; 95% CI: 0.79, 0.99), myocardial infarction (SRR: 0.75; 95% CI: 0.65, 0.93), stroke (SRR: 0.86; 95% CI: 0.75, 0.99), and heart failure (SRR: 0.80; 95% CI: 0.67, 0.95) (159).

An analysis of 49 randomized controlled trials, with durations ranging from one to eight years and included 24,272 participants found that increasing PUFA intake had little no effect on all-cause mortality or cardiovascular disease (risk 7.8 vs 7.6%, risk ratio (RR) 0.98, 95% CI 0.89–1.07). However, it slightly reduces the risk of coronary heart disease (from 14.2 to 12.3%, RR 0.87, 95% CI 0.72 -1.06) and cases of cardiovascular disease events (from 14.6 to 13.0%, RR 0.89, 95% CI 0.79–1.01), and may also slightly reduce the risk of mortality from coronary heart disease (6.6–6.1%, RR 0.91, 95% CI 0.78–1.06) (160).

The INTERMAP (The International Study of Macro- and Micro-nutrients and Blood Pressure) trial showed that ω-3 PUFA intake was inversely related to blood pressure, including in non-hypertensive individuals (161). A meta-analysis of 7 randomized controlled trials showed that EPA + DHA intake lowered blood pressure, most significantly in patients with untreated hypertension (162). In this group of patients, the decrease in systolic blood pressure was 4.51 mm Hg (95% CI =−6.12 to–2.83 mm Hg) and the decrease in diastolic blood pressure was 3.05 mm Hg (95% CI =−4.35 to −1.74 mm Hg). Another study, although it did not confirm the role of ω-6 or ω-3 PUFA intake in changes in blood pressure over time, did show a possible interaction of ω-3 PUFA with the CYP4F2 V433M genotype (163). In a study of 81,579 participants with arterial hypertension, fish oil intake was associated with an 8% reduction in cardiometabolic multimorbidity risk (95% CI 0.89–0.96, *p* < 0.001) and a 10% reduction in all-cause mortality (95% CI 0.85–0.95, *p* < 0.001) (164). Fish oil was associated with a low incidence of sustained ventricular fibrillation episodes in the monkey (165).

A meta-analysis of 13 studies (GISSI-P, JELIS, GISSI-HF, DOIT, SU.FOL.OM3, Alpha Omega, OMEGA, ORIGIN, R&P, AREDS-2, VITAL, ASCEND, REDUCE-IT) including a total of 127,477 patients showed that marine ω-3 PUFAs supplementation significantly reduced risk of myocardial infarction (RR 0.92, 95% CI 0.86–0.99, *p* = 0.020), coronary heart disease (RR 0.95, 95% CI 0.91–0.99, *p* = 0.008) and overall cardiovascular morbidity (RR 0.97, 95% CI 0.94–0.99, *p* = 0.015) (166). A meta-analysis of 38 randomized controlled trials involving 149,051 participants showed that ω-3 PUFAs reduce cardiovascular mortality (RR, 0.93 95% CI 0.88–0.98, *p* = 0.01) and improve cardiovascular outcomes. The risk of non-fatal myocardial infarction (RR, 0.87 95% CI 0.81–0.93, *p* = 0.0001), coronary heart disease events (RR, 0.91 95% CI 0.87–0.96, *p* = 0.0002) was shown to decrease. The reduction in cardiovascular risk was more pronounced with eicosapentaenoic acid monotherapy than with docosahexaenoic acid (167). Importantly, ω-3 PUFAs increased the incidence of atrial fibrillation (OR 1.26 [1.08–1.48]), and eicosapentaenoic acid monotherapy compared with the control group was associated with a higher risk of total bleeding (OR: 1.49 [1.20–1.84]) and atrial fibrillation (OR: 1.35 [1.10–1.66]) (167).

Thus, there is evidence for the promising use of PUFAs in clinical practice due to their diverse functions. At the same time, the data on the clinical efficacy of PUFAs vary.

While earlier studies showed predominantly positive effects, more recent studies have shown no significant clinical effects of PUFA supplementation and, in some cases, negative results. In a large cohort of patients (12,513) with multiple cardiovascular risk factors, daily treatment with ω-3 PUFAs did not reduce cardiovascular mortality or morbidity compared with placebo (olive oil) [11,7% vs 11,9% (RR 0.97, 95% CI 0.88–1.08, *p* = 0.58)] (168). Treatment with ω-3 PUFAs for 3 months did not improve endothelial function in patients with type 2 diabetes and very high cardiovascular risk (169). Consumption of ω-3 PUFAs at a dose ≤ 1.8 g/d for 12 months also did not improve endothelial function in healthy adults (170).

In addition to examining the role of ω-3 PUFAs, there is increasing interest in finding the optimal ratio of ω-6/ω-3 PUFAs (171). The significance of ω-6 PUFAs for cardiovascular disease has also been the subject of much research over a long period of time (172). Linoleic acid is an essential ω-6 fatty acid because it cannot be synthesized by humans (173). It is the most common PUFAs in the human diet, as significant amounts of linoleic acid are present in vegetable oils.

Linoleic acid has been shown to reduce LDL cholesterol levels (174). And higher intake of linolenic acid was associated with a lower risk of atherosclerotic plaques in the carotid arteries (175). A meta-analysis of 30 prospective studies involving 68,659 participants found that higher concentrations of linolenic acid were significantly associated with lower risks of overall cardiovascular disease (hazard ratio (HR) 0.93, 95% CI 0.88–0.99), cardiovascular mortality (HR 0.78, 95% CI 0.70–0.85), and risks of ischemic stroke (HR 0.88, 95% CI 0.79–0.98) (176). An analysis of 13 cohort studies with a total of 310,602 participants showed that dietary intake of linoleic acid was inversely associated with the risk of coronary heart disease in a dose-dependent manner (177). On the other hand, evaluation of reconstructed data from the Sydney Dietary Heart Study and an updated meta-analysis showed that dietary linoleic acid use from safflower oil increased the risk of death from coronary heart disease and cardiovascular disease (178). The study, which included 20,000 middle-aged men and women in the Netherlands, found that linoleic acid intake was not associated with the ratio of total cholesterol to HDL cholesterol, and no association was found between linoleic acid intake and the incidence of CHD over a 10-year follow-up period (179).

Cost-effectiveness analysis of long-term treatment with ω-3 PUFAs after myocardial infarction showed comparable results with other drugs for secondary prevention after myocardial infarction (180). Another five-country study showed that adding highly concentrated ω-3 PUFAs to standard treatment for secondary prevention of myocardial infarction was cost-effective compared with standard treatment alone (181). Combination therapy with eicosapentaenoic acid and statins showed acceptable cost-effectiveness for secondary prevention of cardiovascular disease in patients with hypercholesterolemia in Japan (182). Adding ω-3 PUFAs to optimal drug therapy for patients with heart failure may also be cost-effective (183). Treatment with icosapent etyl has been associated with both higher costs and higher benefits (184). Omacor has also been shown to be cost-effective as a prophylactic after myocardial infarction, both after 4 years and during life (185).

Thus, the clinical significance of PUFAs for cardiovascular disease is the subject of research and debate, but all questions have not been answered to date.

### The significance of polyunsaturated fatty acids in coronary heart disease

A significant number of studies have analyzed the diagnostic, prophylactic and therapeutic value of PUFAs. It has been shown that patients with CHD have lower levels of ω-6 and ω-3 PUFAs, especially eicosapentaenoic acid, in the blood (186). In a study including 2,692 American adults aged 74 ± 5 years, high individual and overall levels of circulating ω-3 PUFAs (eicosapentaenoic acid, docosapentaenoic acid, and docosahexaenoic acid) were found to be associated with lower overall mortality, especially mortality from CHD (187).

In the GISSI-Prevenzione trial, dietary supplements with ω-3 PUFAs in patients who had had a myocardial infarction resulted in clinically important effects, providing the basis for their therapeutic use (188). However, in a mortality risk assessment study of 3,114 men under age 70 with angina who ate fatty fish or fish oil, no beneficial effect was shown. Moreover, men who ate this diet had a higher risk of cardiac death (189). In a multicenter trial including 4,837 patients aged 60–80 years (78% men) who had had a myocardial infarction in addition to current antihypertensive, antithrombotic, and lipid-modifying therapies and received various combinations of marine ω-3 fatty acids eicosapentaenoic acid and docosahexaenoic acid and plant alpha linolenic acid and placebo. The results of the study showed no significant reduction in the incidence of serious cardiovascular complications when low-dose eicosapentaenoic acid-docosahexaenoic acid or alpha-linolenic acid were taken (190).

The JELIS (Japan EPA Lipid Intervention Study) evaluated the effect of eicosapentaenoic acid on major coronary events in 18,645 patients with hypercholesterolemia. It found that unstable angina and non-fatal coronary events were significantly reduced in the eicosapentaenoic acid group. There were no differences in sudden cardiac death and coronary death (191).

In the OMEGA-REMODE randomized clinical trial, administration of high-dose omega-3 fatty acids for 6 months to patients undergoing acute myocardial infarction was associated with a reduction in adverse left ventricular remodeling as well as non-infarct myocardial fibrosis and serum biomarkers of systemic inflammation (192). More recently, genetic profiling of fatty acid desaturase 2 (FADS2) polymorphisms has been shown to identify patients who may benefit from high-dose omega-3 PUFAs for cardiac remodeling after acute myocardial infarction (193).

An analysis of 86 randomized controlled clinical trials (162,796 participants) that lasted at least 12 months showed little or no effect of increasing omega-3 PUFA intake on all-cause mortality, cardiovascular mortality, and cardiovascular events. However, ω-3 PUFA intake may marginally reduce the risk of mortality from coronary heart disease and complications of coronary heart disease (194).

A recent meta-analysis including 14 randomized controlled clinical trials (135,291 patients) showed that taking ω-3 PUFAs reduced the risk of major adverse cardiovascular events, cardiovascular death, and myocardial infarction, but had no significant effect on all-cause mortality (195). And taking ω-3 PUFAs can prevent myocardial infarction regardless of the stage of CHD (195).

Thus, data from various studies demonstrate different clinical efficacy of ω-3 PUFAs in the prevention and treatment of coronary heart disease, which requires additional research.

### The significance of polyunsaturated fatty acids in peripheral arterial disease

Peripheral artery disease (PAD) is another important clinical problem associated with endothelial dysfunction, inflammation, and atherosclerosis. PAD was found to be deficient in ω-3 PUFAs in the erythrocyte membrane and to have a lower ratio of eicosapentaenoic acid to arachidonic acid (196). In a randomized, placebo-controlled, double-blind trial in 70 patients with peripheral arterial disease, endothelial function was shown to improve without affecting pain-free walking distance after 3 months of treatment with 4 grams of ω-3 PUFAs (197).

In another study, administration of ω-3 PUFAs for 3 months to patients with PAD, caused a marked improvement in endothelial function but had no effect on inflammatory status (198). The OMEGA-PAD I Trial, which evaluated the effect of short-term high-dose ω-3 PUFAs, showed no difference in endothelial function between the treatment and placebo groups. However, there was an increase in production of markers of specialized pro-resolving mediators, in patients with PAD (199). The OMEGA-PAD II Trial, which was conducted to evaluate the effect of high-dose oral ω-3 PUFA supplementation for 3 months on inflammation, endothelial function, and walking ability in patients with PAD, showed increased SPMs in the plasma of patients with PAD (200).

### Significance of polyunsaturated fatty acids in atrial fibrillation

Atrial fibrillation (AF) is an urgent medical problem due to its high prevalence and impact on prognosis. This information strengthens the understanding of the importance of atrial fibrillation prevention.

Increased serum concentrations of long-chain ω-3 PUFAs have been shown to be associated with a lower risk of AF. A prospective population-based study of coronary heart disease risk factors, including 2,174 men followed for 17.7 years, showed that increased serum concentrations of long-chain ω-3 PUFAs may protect against AF. Serum docosahexaenoic acid concentration showed the greatest effect on the risk of AF (201). Another study of 3,326 men and women aged ≥ 65 years found that higher plasma levels of total long-chain ω-3 PUFAs and docosahexaenoic acid were associated with a lower risk of AF (202).

Animal studies have provided evidence for a positive role of ω-3 PUFAs for the prevention of atrial fibrillation. Eicosapentaenoic acid prevented atrial fibrillation associated with heart failure in a rabbit model (203). Dietary ω-3 fatty acids reduced atrial infiltration by inflammatory cells and reduced the inducibility of postoperative atrial fibrillation in a canine cardiac surgery model (204).

However, many clinical studies have failed to confirm the positive role of PUFA supplementation for the prevention of AF.

The STRENGTH randomized clinical trial included 13,078 patients with high cardiovascular risk treated with statins. Adding omega-3 eicosapentaenoic acid and docosahexaenoic acid to conventional background therapy compared with corn oil did not result in a significant difference in a composite outcome of major adverse cardiovascular events. However, the ω-3 PUFA group (24.7%) had a higher incidence of gastrointestinal adverse events compared with patients receiving corn oil (14.7%). Interestingly, patients taking ω-3 PUFA had a higher incidence of first-time atrial fibrillation (2.2% vs. 1.3%) compared with corn oil (205).

Another trial, including 25,119 participants aged 50 years or older, evaluated the effect of long-term ω-3 fatty acid (eicosapentaenoic acid and docosahexaenoic acid) and vitamin D supplementation on the incidence of AF. The results of the study showed no significant difference in the risk of AF over a follow-up period of more than 5 years among the groups taking eicosapentaenoic acid – docosahexaenoic acid or vitamin D3 compared with placebo (206).

In the REDUCE-IT trial, patients who received highly purified ethyl ester of eicosapentaenoic acid (icosapent ethyl) had a higher risk of hospitalization for AF compared with placebo (3.1% versus 2.1%) over a median of 4.9 years (207). Moreover, the risk of ischemic events, including cardiovascular death, was significantly lower among patients taking icosapent ethyl than among those receiving placebo (207).

In the OMEMI (Omega-3 Fatty acids in Elderly with Myocardial Infarction) randomized clinical trial, elderly patients after myocardial infarction received omega-3 fatty acids (a combination of eicosapentaenoic acid and docosahexaenoic acid) or corn oil for 2 years. Results showed no reduction in clinical events, whereas 7.2% of patients in the omega-3 fatty acid group versus 4.0% in the corn oil group developed atrial fibrillation (208). A secondary analysis of the OMEMI trial showed that a greater increase in eicosapentaenoic acid during treatment was associated with a higher risk of first-onset AF (209).

The Danish cohort study (*n* = 54,737) showed no association between ω-3 or ω-6 PUFAs and atrial fibrillation (210). A randomized, double-blind, multicenter trial of ω-3 PUFAs showed no efficacy in preventing arrhythmia recurrence after electrical cardioversion of chronic persistent atrial fibrillation (211).

These data suggest that ω-3 fatty acid intake does not show a reduced risk of atrial fibrillation. In addition, in another large trial [The Omega-3 Fatty Acids for Prevention of Post-operative Atrial Fibrillation (OPERA)] in which patients received perioperative ω-3-PUFA supplementation before heart surgery found no reduction in the risk of postoperative AF compared with placebo (212).

A meta-analysis of randomized controlled trials showed that preoperative PUFA treatment had no effect on the incidence of postoperative atrial fibrillation in patients undergoing open-heart surgery (213). In addition, a recent systematic review and meta-analysis of 7 studies involving 81,210 patients showed that long-term supplementation with marine ω-3 PUFAs was associated with an increased risk of atrial fibrillation (HR 1.25, 95% CI 1.07-1.46, *p* = 0.013). The increased risk of atrial fibrillation was most significant with omega-3 PUFA supplementation at doses > 1 g/day (214).

The findings suggest that other studies are needed to evaluate the molecular mechanisms linking the incidence of AF to PUFAs intake.

It should be noted that ω-3 PUFAs demonstrate both antiarrhythmic and proarrhythmic effects through their action on different mechanisms (120). Given that different arrhythmogenic mechanisms are characteristic of different patient subgroups, the effects of ω-3 PUFAs on different types of arrhythmias require further study (120).

### The importance of polyunsaturated fatty acids in heart failure

Heart failure is a complex syndrome that is the final outcome of the progression of many cardiovascular diseases, such as coronary heart disease and arterial hypertension. Heart failure is an important cause of reduced quality of life, hospitalizations, and death. The rate of progression of heart failure depends on many factors and is an important target for therapeutic interventions, primarily regarding its causes. Because of the great medical and social significance of heart failure, new effective therapies are required. Numerous studies have investigated the possible benefits of taking ω-3 PUFAs as a means of preventing and treating heart failure.

In the MESA (Multi-Ethnic Study of Atherosclerosis) trial, in 6,562 participants aged 45–84 years, followed for 13.0 years, higher plasma eicosapentaenoic acid levels were significantly associated with a reduced risk of heart failure in both patients with reduced and preserved ejection fractions (215). Over a follow-up period of 15.6 years, higher plasma levels of ω-3 fatty acids were shown to be associated with fewer long-term cardiovascular events. And the absolute reduction in the incidence of cardiovascular disease with higher levels of ω-3 PUFAs was more evident with higher coronary artery calcium levels (216). In addition, another study showed that the eicosapentaenoic acid/arachidonic acid ratio in the blood was an independent predictor of cardiac mortality in patients with heart failure (217). And the most pronounced effect of the eicosapentaenoic acid/arachidonic acid ratio on cardiac mortality was among patients shown to be taking statins.

In a recent meta-analysis that included 13 studies, it was shown that high dietary intake of ω-3 PUFAs was associated with a lower risk of heart failure in 8 of those studies, which included more than 300,000 participants (218). Another six studies involving 17,163 participants showed an association of higher concentrations of circulating ω-3 PUFAs with a lower risk of heart failure (218). In addition, high circulating concentrations of docosahexaenoic acid have been shown to be associated with a reduced risk of heart failure, while no significant association was found for eicosapentaenoic and docosapentaenoic acids (218).

Interestingly, eicosapentaenoic acid, but not docosahexaenoic acid, prevents fibrosis in pressure overload-induced heart failure, due to a potential role of the free fatty acid receptor 4 (Ffar4) linked to G-protein (219). Eicosapentaenoic acid prevents cardiac fibrosis and diastolic dysfunction in heart failure with preserved ejection fraction signaling through Ffar4 (220). The cardioprotective mechanisms of eicosapentaenoic acid in heart failure are related to its ability to activate NRF2 (nuclear factor erythropoietin 2 related factor 2), Ffar4 receptor (free fatty acid receptor 4), or GPR120 fibroblast receptors. This activation inhibits cardiac fibrosis and protects the heart from heart failure (221). NRF2 is regulated by specialized pro-resolving mediators derived from docosahexanoic acid and eicosapentaenoic acid (222). NRF2 is a transcription factor that participates in the control of oxidative stress, thus providing myocardial protection against the occurrence of fibrosis (222). In addition, Nrf2 was shown to suppress TNF-α-induced monocyte chemoattractant protein (MCP)-1 and VCAM-1 mRNA and protein expression in a dose-dependent manner and inhibited TNF-α-induced adhesion of human monocytic U937 cells to human aortic endothelial cells (HAEC) (223).

These data suggest that the molecular mechanisms and clinical effects of various PUFAs need to be better understood. Clinical, and corresponding pathophysiological differences in heart failure forms, should be considered in studies to evaluate the role of different PUFAs.

## Conclusion

The problem of cardiovascular disease remains one of the most urgent topics in modern medicine. A large number of patients, a significant impact on the quality of life, ability to work and prognosis, is a serious economic and social burden for patients, their families, society and health care systems in many countries. The importance of the problem is also underscored by the fact that a significant number of cardiovascular diseases today have limited therapeutic prospects. This and other information reinforces the importance of preventive measures that could reduce the risks of cardiovascular complications. ω-3 PUFAs have been considered for a relatively long time as promising agents for the prevention and treatment of several cardiovascular diseases.

Accumulated evidence suggests that the cardioprotective properties of ω-3 PUFAs are associated with their anti-inflammatory effects, participation in the regulation of the biophysical properties of plasma membranes, modification of signaling pathways and cardiac ion channels, and improvement of endothelial function.

Despite the large number of studies conducted, many of the data obtained are contradictory and, when analyzed in combination, do not give confidence in the full therapeutic efficacy of ω-3 PUFAs (Table 1). Moreover, analysis of many recent studies has shown that the benefit of PUFAs in preventing cardiovascular events may be exaggerated. In particular, an increased risk of atrial fibrillation with PUFAs has been shown.

**TABLE 1:** Results of studies on the clinical effectiveness of PUFAs.

Study/ ClinicalTrials.gov number	Year of publication	Purpose of the study	Sample size	Result	References
GISSI-Prevenzione trial	1999	The effect of dietary supplements with ω-3 PUFA and vitamin E after myocardial infarction was studied	11,324 patients with recent (< or = 3 months) myocardial infarction	Dietary supplementation with ω-3 PUFAs resulted in clinically significant effects	(188)
INTERMAP (The International Study of Macro- and Micro-nutrients and Blood Pressure)/NCT00005271	2007	The effect of ω-3 PUFA intake on blood pressure was evaluated	4,680 men and women ages 40–59 from 17 population-based samples	Consumption of ω-3 PUFAs was inversely related to blood pressure, including in non-hypertensive individuals	(161)
JELIS (Japan EPA Lipid Intervention Study)/NCT00231738	2007	The effect of eicosapentaenoic acid on major coronary events in patients with hypercholesterolemia was evaluated	18,645 patients with a total cholesterol level of 6.5 mmol/L	Patients with a history of coronary heart disease treated with eicosapentaenoic acid had a 19% reduction in major coronary events	(191)
–	2009	The association between ω-3 PUFAs in serum (eicosapentaenoic acid, docosapentaenoic acid, and docosahexaenoic acid) and the risk of atrial fibrillation in men	2,174 men between the ages of 42 and 60	Increased serum concentrations of ω-3 PUFAs may protect against atrial fibrillation. The greatest effect was on the serum concentration of docosahexaenoic acid.	(201)
Alpha Omega Trial NCT00127452	2010	The effects of marine ω-3 PUFAs [eicosapentaenoic acid (EPA) and docosahexaenoic acid (DHA)] and plant-derived alpha-linolenic acid (ALA) on the incidence of cardiovascular events in patients with myocardial infarction	4,837 patients aged 60–80 years (78% men) who had a myocardial infarction and received modern antihypertensive, antithrombotic, and lipid-modifying therapies	Administration of low doses of EPA-DHA or ALA did not significantly reduce the incidence of serious cardiovascular complications in patients who had myocardial infarction and were receiving modern antihypertensive, antithrombotic, and lipid-modifying therapies	(190)
The Omega-3 Fatty Acids for Prevention of Post-operative Atrial Fibrillation (OPERA) NCT00970489	2012	Study of ω-3 PUFA administration for the prevention of postoperative atrial fibrillation	1,516 patients who underwent heart surgery	perioperative ω-3 PUFA supplementation compared with placebo did not reduce the risk of postoperative atrial fibrillation	(212)
ORIGIN Clinical Trial, NCT00069784	2012	The effect of ω-3 PUFA supplementation on cardiovascular outcomes in patients with dysglycemia was evaluated	12,536 patients with a high risk of cardiovascular events and impaired fasting glucose, impaired glucose tolerance, or diabetes	Daily intake of 1 g of ω-3 PUFAs did not reduce the incidence of cardiovascular events in patients at high risk of cardiovascular events	(227)
The OMEGA-PAD I Trial/NCT01310270	2015	The effect of fish oil intake on endothelial function in patients with peripheral arterial disease was evaluated	80 patients	Short-term administration of high doses of fish oil did not improve endothelial function, but it did improve serum triglyceride levels and increased mediator production from ω-3 PUFAs in patients with PAD	(199)
OMEGA-REMODE Randomized Clinical Trial/NCT00729430	2016	The effect of ω-3 PUFA ethyl esters on left ventricular remodeling after acute myocardial infarction was evaluated	Patients with acute myocardial infarction: high-dose ω-3 fatty acids (*n* = 180) or placebo (*n* = 178)	treatment with high-dose ω-3 PUFAs was associated with a reduction in adverse left ventricular remodeling, non-infarcted myocardial fibrosis, and serum biomarkers of systemic inflammation	(192)
The Danish cohort study	2017	A study of the relationship between PUFAs and the risk of atrial fibrillation	54,737 patients	No association was found between ω-3 and ω-6 PUFA intake and atrial fibrillation	(210)
ASCEND/NCT00135226	2018	Analysis of the effects of ω-3 fatty acid supplementation in diabetes mellitus.	15,480 patients with diabetes but no signs of atherosclerotic cardiovascular disease	Among diabetic patients with no evidence of cardiovascular disease, there was no significant difference in the risk of serious vascular events between those who were given n-3 PUFA supplements and the placebo group	(228)
REDUCE-IT/NCT01492361	2018	Assessment of cardiovascular risk while taking Icosapent Ethyl	8179 hypertriglyceridemic patients	The risk of ischemic events, including cardiovascular death, was significantly lower among patients who received icosapent ethyl	(207)
MESA (Multi-Ethnic Study of Atherosclerosis)/NCT00005487	2019	The aim of the study was to determine whether plasma eicosapentaenoic acid (EPA) content was associated with a decreased risk of primary heart failure events	6,562 participants between the ages of 45 and 84	Higher plasma levels of ω-3 PUFAs were associated with less long-term cardiovascular disease	(202)
The OMEGA-PAD II Trial/NCT01979874	2019	Evaluation of the effects of fish oil supplementation in patients with peripheral arterial disease	24 patients with peripheral arterial disease	Fish oil increases SPM in the plasma of patients with PAD	(200)
The VITamin D and OmegA-3Trial (VITAL)/NCT01169259	2019	The effect of vitamin D3 and marine ω-3 PUFA supplementation in the primary prevention of cardiovascular disease was studied	25,871 men aged ≥ 50 and women aged > 55	ω-3 PUFA supplements did not reduce risk of major cardiovascular events	(229)
STRENGTH/NCT02104817	2020	A study of the effects of high-dose ω-3 fatty acids and corn oil on major adverse cardiovascular events in patients with high cardiovascular risk	13,078 patients with high cardiovascular risk receiving statins	Patients taking ω-3 PUFAs had a higher rate of first-time atrial fibrillation (2.2% vs. 1.3%) compared with corn oil	(205)
VITAL Rhythm Study/NCT02178410	2021	A study of the effect of marine ω-3 PUFAs (EPA and DHA) and vitamin D supplementation on the occurrence of atrial fibrillation	25,119 women and men aged 50 years or older with no previous cardiovascular disease or atrial fibrillation	Results do not support the benefit of marine ω-3 PUFAs or vitamin D3 in adults for the prevention of atrial fibrillation	(206)
OMEMI (Omega-3 Fatty acids in Elderly with Myocardial Infarction) NCT01841944	2021	The effects of ω-3 PUFAs supplementation in elderly patients after myocardial infarction were investigated	1,014 patients with a mean age of 75 ± 3.6 years with recent myocardial infarction who took 1.8 g of ω-3 PUFAs daily for 2 years	No reduction in clinical events was found in elderly patients	(208)

At the same time, it should be noted that many studies do not take into account the influence of concomitant factors, including physical activity levels and patients’ attitudes toward treatment. In addition, the importance of other nutritional factors, including other oils taken by patients, which may have influenced the outcome of the studies, must be considered.

Interestingly, salmon (*Salmo salar L*), which is a good dietary source of PUFAs, itself suffers from coronary atherosclerosis (224, 225). And a diet enriched in cholesterol led to a worsening of lipid indices and progression of lesions (226). These and other data underscore the need for a comprehensive evaluation of various factors.

Thus, PUFAs are involved in the regulation of a large number of mechanisms that have complex cross-linkages and are associated with cardiovascular disease. This requires new research that would expand the boundaries of our understanding of the processes that occur in the vascular wall and how these processes can be effectively influenced.

## Author contributions

SK and AK contributed to the conception of research and wrote the manuscript. SK contributed to the revision of the manuscript. Both authors approved the final manuscript for submission.
